# Graft-Versus-Host Disease: A Surge of Developments

**DOI:** 10.1371/journal.pmed.0040198

**Published:** 2007-07-10

**Authors:** Stanley R Riddell, Frederick R Appelbaum

## Abstract

Stanley Riddell and Frederick Appelbaum review progress in preventing graft-versus-host disease following allogeneic hematopoietic cell transplantation for malignancies or other life-threatening blood diseases.

## Introduction

This year approximately 20,000 individuals will receive an allogeneic hematopoietic cell transplant (HCT) as treatment for a malignant, or life-threatening non-malignant, hematopoietic disease. The process of HCT generally begins with administration of a preparative regimen to eradicate the underlying disease and immunosuppress the patient in order to prevent rejection of the subsequently transfused hematopoietic stem cells. Following HCT, donor T cells transplanted with or developing from the hematopoietic stem cells react with cells of the human leukocyte antigen (HLA)-matched but genetically non-identical host, providing a beneficial graft-versus-tumor (GVT) response but also resulting in possibly life-threatening graft-versus-host disease (GVHD). The manifestations of GVHD vary over its course. Acute GVHD usually appears within several weeks of HCT and is characterized by a diffuse maculopapular rash, mucosal inflammation causing crampy abdominal pain and diarrhea, and elevated liver function tests (Figure 1). GVHD that first appears or persists more than three months after allogeneic HCT is termed chronic GVHD and resembles a chronic autoimmune disorder. Patients frequently develop lichen planus skin lesions, ocular and oral sicca, obliterative bronchiolitis, and hepatic abnormalities resembling primary biliary sclerosis.

**Figure 1:**
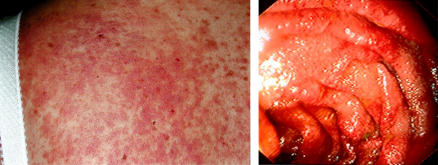
Clinical Appearance of Acute Graft-Versus-Host Disease Involving the Skin and the Upper Intestinal Mucosa Left panel: The diffuse erythematous maculopapular rash typical of acute GVHD. Right panel: an endoscopic view of the edematous, reddened, friable gastrointestinal mucosa seen in a patient with acute GVHD.

If no immunosuppression is given after allogeneic HCT, life-threatening or fatal GVHD inevitably develops. The first successful application of allogeneic HCT to treat human leukemia in the early 1970s was made possible by the use of methotrexate, administered early after transplantation as prophylaxis against GVHD [1]. In the mid-1980s, prospective randomized trials were performed demonstrating that a combination of a calcineurin inhibitor (cyclosporin or tacrolimus) plus methotrexate was superior to either agent alone in preventing acute GVHD [2,3], and such combinations remain the standard of care today. Despite such prophylaxis, approximately 50% of patients receiving HCT will develop acute GVHD sufficiently severe to require additional immunosuppression, usually in the form of a corticosteroid, and approximately 50% of patients will develop chronic GVHD requiring continued immunosuppression for up to several years. The majority of patients eventually develop tolerance, and immunosuppression can be completely withdrawn in these cases, but 10%–20% of recipients of HLA-matched hematopoietic cell transplants will die of refractory GVHD or of opportunistic infections associated with its prevention or treatment, and the mortality rate increases with increasing donor–recipient HLA disparity.

An elusive goal of research has been to find ways to prevent GVHD without dramatically increasing other transplant complications. Most clinical studies to date have focused on the use of alternative immunosuppressants or removal of T cells from the donor stem cell source. More intensive post-transplant immunosuppressive regimens and T cell depletion are both capable of dramatically reducing the incidence and severity of GVHD, but do so at the cost of an increased incidence of fatal post-transplant infections and tumor recurrence. Increased graft rejection may also occur if donor T cells are removed from the donor stem cell graft, because the reaction of these cells against residual host immune cells contributes to engraftment. More intensive preparative regimens can overcome this problem but are associated with increased toxicity. More potent post-transplant immunosuppressive regimens and T cell depletion suppress or remove immunocompetent cells responsible for protection against many pathogens as well as cell populations that contribute to the beneficial GVT response. Accordingly, in retrospective analyses and prospective randomized trials, neither more intensive post-transplant immunosuppression nor T cell depletion has been demonstrated to improve survival following allogeneic HCT [4]. While it is the case that fewer patients succumb to GVHD and associated complications today than did a decade ago, this is largely due to improvements in HLA-typing technology and supportive care measures rather than advances in the direct prevention and treatment of GVHD. Recent clinical and animal model studies have provided several novel and surprising insights into the biology of GVHD and provide exciting new directions for strategies that may prevent GVHD without increasing complications.

## Haplotype Matching

One area of progress, as noted above, has been in HLA-typing technology and donor selection. Historically, HLA typing was conducted using serologic methods, but with the advent of the polymerase chain reaction assay in the 1980s, it became possible to perform molecular typing of donor and recipient. When patients previously transplanted from serologically matched donors were retrospectively analyzed using molecular typing, approximately 30% were found to be mismatched with the donor for one or more alleles, and such mismatching was shown to lead to more GVHD and poorer survival [5]. Thus, molecular typing of the HLA locus has become the standard of care. Even with the use of molecular typing to identify fully HLA-matched donors, unrelated donor transplants continue to be associated with more GVHD than seen with HLA-matched sibling transplants. Using a novel technique that allows for the typing of individual DNA strands, Petersdorf et al. have now shown that among allele-matched unrelated donor–recipient pairs, those that shared the same physical linkage of HLA-A, -B, and -DRB1 were much less likely to develop severe GVHD (Figure 2) [6]. These results imply that other unidentified transplantation antigens exist within the same genetic region as HLA, and offer a method for improved selection among HLA-matched unrelated donors.

**Figure 2:**
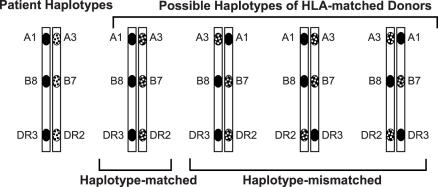
Schematic Illustration of the HLA Haplotypes of a Patient and 4 HLA Phenotypically Identical Potential Donors Data suggest that the use of the single haplotype-matched donor reduces acute graft-versus-host disease.

## Genetic Profiling

The pathogenesis of GVHD involves the expansion and differentiation of donor T cells reacting in peripheral lymphoid tissues against host antigen-presenting cells that display disparate minor histocompatibility antigens. These antigen-presenting cells are activated as a consequence of tissue injury and the resulting release of proinflammatory cytokines [7]. Polymorphisms in cytokine genes between donor and recipient may influence the host inflammatory response to tissue injury and the severity of GVHD. The interleukin-10 (IL-10) pathway is the most extensively studied, and specific polymorphisms in the recipient *IL-10* promoter region as well as in the donor *IL-10* receptor beta gene have each been found to be associated with a lower risk of GVHD and non-relapse mortality [8,9]. Polymorphisms in other immune regulatory genes, including those encoding interleukin-6, interferon gamma, and tumor necrosis factor-alpha, have also been suggested to influence the development of GVHD [10].

**Five Key Papers in the Field**

**Ferrara et al., 2006** [7] This review discusses the involvement of cytokine networks and links activation of the innate and adaptive immune system with the pathogenesis of GVHD.

**Wagner et al., 2005** [4] A multicenter randomized trial of T cell depletion versus methotrexate and cyclosporine for graft-versus-host disease prophylaxis shows that T cell depletion is effective for preventing GVHD but fails to improve survival because of increased relapse and infection.

**Anderson et al., 2003** [12] Stem cell grafts devoid of naïve T cells but containing memory T cells do not cause GVHD and improve immune reconstitution in a murine allogeneic hematopoietic cell transplant model.

**Dickinson et al., 2002** [22] This paper describes an elegant in situ model of human skin GVHD and demonstrates that ubiquitously expressed minor histocompatibility antigens are primary targets of GVHD.

**Bonnet et al., 1999** [23] The first direct demonstration that CD8^+^ minor histocompatibility antigen–specific T cells recognize and eliminate human acute myeloid leukemia stem cells.

In an effort to provide a more global assessment of whether a donor is likely to induce GVHD, Baron et al. have examined gene expression profiles of CD4 and CD8 T cells from donors and report that it is possible to segregate donors into those likely to cause GVHD and those who are relatively safe [11]. If this ability to discriminate between strong and weak alloresponders is verified in further studies, these findings could have important implications for donor selection.

## Donor Graft Manipulation

Stem cell grafts contain distinct functional and phenotypic subsets of T cells, including antigen-inexperienced naïve T cells (T_N_), antigen-experienced memory T cells, (T_M_), and regulatory T cells (T_REG_). Recent studies have begun to dissect the contribution of these individual T cell subsets to GVHD and have identified opportunities for more refined manipulation of the T cell content of stem cell grafts that may reduce GVHD without the severe T cell deficiency associated with complete depletion (Figure 3). For example, the selective depletion of T_N_ from allogeneic stem cell grafts abrogated GVHD in both CD4- and CD8-dependent multiple minor histocompatibility antigen–mismatched mouse models, and the remaining T_M_ provided reconstitution of immunity to pathogens [12,13]. Human T_N_ and T_M_ can also be distinguished based on phenotype— T_N_ are CD45RA+ and CD62L+, while T_M_ are CD45RO+ and either CD62L+ or CD62L-, and emerging data suggest that alloreactivity for minor histocompatibility antigens is predominantly contained in the T_N_ repertoire subset [14]. The human T_M_ comprises less than 1% of the overall T cell receptor diversity and consists of large numbers of T cells specific for cytomegalovirus, Epstein Barr virus, and other pathogens that cause opportunistic infections in HCT recipients [15]. Thus, unless the donor has been previously sensitized to recipient minor histocompatibility antigens (which would convert alloreactive naïve T cells to the memory pool), transplants using stem cells depleted of naïve T cells could reduce or eliminate GVHD while preserving the transfer of memory T cells to common infectious agents. Such transplants would overcome a major limitation of transplantation using complete T cell depletion. The recognition that donor *CD4* ^+^ *CD25* ^+^ *Foxp3* ^+^ T_REG_ cells suppress T cell responses in vitro and in vivo suggests another attractive approach to donor graft manipulation for preventing GVHD. The importance of T_REG_ in GVHD is supported by murine studies showing that their depletion from stem cell grafts exacerbates GVHD and that the infusion of additional T_REG_ at the time of HCT reduces lethal GVHD, apparently by limiting the initial activation of alloreactive T cells in lymph nodes [16,17]. Clinical studies have suggested that stem cell grafts from donors with higher numbers of T_REG_ confer a lower risk of GVHD [18], and efforts are in progress to isolate and expand populations of human T_REG_ that might be used to supplement stem cell grafts and abrogate GVHD [19].

**Figure 3:**
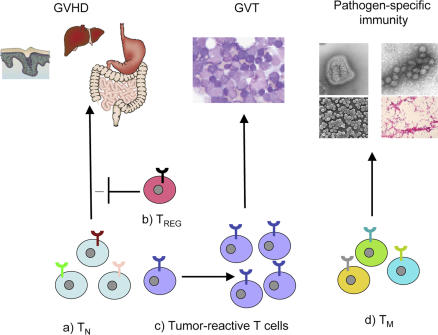
Selective Manipulation of T Cell Subsets in Allogeneic Stem Cell Grafts to Reduce GVHD while Retaining GVT and Pathogen-Specific Immunity Strategies being developed to modify the T cell content of allogeneic stem cell grafts include: a) depletion of the T_N_ subset of cells that contain the repertoire of T cells capable of recognizing minor histocompatibility antigens expressed on skin, gastrointestinal, and hepatic tissues; b) expanding T_REG_ cells that interfere with activation of alloreactive T cells to augment the stem cell graft; c) isolation and expansion of tumor-reactive T cells from naïve T cell progenitors for adoptive immunotherapy to augment the GVT effect; and d) retention of T_M_ cells in the graft to restore protective T cell immunity to pathogens.

## Segregation of GVHD from GVT

Although these new approaches to allogeneic HCT are likely to reduce the severity of GVHD, an important concern for patients undergoing HCT for a malignant disease is whether reducing GVHD might increase the risk of tumor recurrence. Like GVHD, GVT is the result of donor T cells reacting with disparate minor histocompatibility antigens, and elimination of GVHD would seem almost certain to diminish the GVT effect. Elucidation of the molecular structure, HLA restriction, and tissue expression of human minor histocompatibility antigens, and the identification of non-polymorphic leukemia-associated antigens that can be recognized by T lymphocytes offers the exciting prospect that targeted T cell therapy after HCT might selectively augment GVT activity [20]. An increasing number of minor histocompatibility antigens have now been molecularly characterized, and novel mechanisms of polypeptide processing have been uncovered [21]. Several minor histocompatibility antigens are expressed in both normal and malignant hematopoietic cells of the recipient, but not in epithelium [22]. Thus, donor T cells reactive with such tissue-restricted antigens will target recipient hematopoietic and leukemic cells without damaging non-hematopoietic tissues or engrafting donor hematopoietic progenitors. Techniques for cloning minor histocompatibility antigen–specific T cells have been developed, and these T cell clones can eliminate primary human leukemias in immunodeficient mice [23]. Pilot studies in which cloned donor T cells reactive with minor histocompatibility antigens have been adoptively transferred to treat patients with post-transplant disease recurrence have demonstrated the principle that GVT and GVHD can be segregated based on the tissue expression of the target antigen [24]. The feasibility of this strategy is advancing with refinements in the culture methods used for isolating effector T cells from naïve T cell precursors and programming these effector cells for GVT activity. The identification of candidate leukemia antigens that are not derived from polymorphic proteins provides additional targets for T cell therapy or vaccination that may be broadly applied [25]. Thus, strategies to abrogate GVHD and its complications need not be associated with loss of the GVT effect, but may instead employ targeted immunotherapy to reduce the intensity and duration of post-grafting immunosuppression while augmenting GVT activity.

## Conclusion

The human graft-versus-host reaction continues to both fascinate and frustrate clinical investigation with its lack of predictability, possibly lethal toxicity, but potentially lifesaving anti-tumor effects. The ability to select optimal donors based on improved HLA-typing technologies and better understanding of non-HLA contributions to immune reactivity seems at hand, and should substantially reduce the risk of developing severe GVHD. Clinical studies of donor graft manipulation removing T_N_ subsets, retaining T_M_ subsets, and possibly supplementing T_REG_ subsets are just being initiated. In the long term, perhaps the most exciting potential lies in the segregation of GVHD from the GVT reaction based on increased recognition of minor histocompatibility antigens exclusively expressed on normal versus malignant hematopoietic tissues. Whether these new advances will lead to the hoped-for clinical victories will become apparent in the next few years.

## Abbreviations

GVHD: graft-versus-host disease
GVT: graft-versus-tumor
HCT: hematopoietic cell transplant
HLA: human leukocyte antigen
IL: interleukin
T_M_: memory T cells
T_N_: naïve T cells, T_REG_, regulatory T cells
